# Procalcitonin reflects bacteremia and bacterial load in urosepsis syndrome: a prospective observational study

**DOI:** 10.1186/cc9328

**Published:** 2010-11-17

**Authors:** Cees van Nieuwkoop, Tobias N Bonten, Jan W van't Wout, Ed J Kuijper, Geert H Groeneveld, Martin J Becker, Ted Koster, G Hanke Wattel-Louis, Nathalie M Delfos, Hans C Ablij, Eliane MS Leyten, Jaap T van Dissel

**Affiliations:** 1 Department of Infectious Diseases, Leiden University Medical Center, Albinusdreef 2, 2333 ZA, Leiden, The Netherlands; 2 Department of Internal Medicine, Bronovo Hospital, Bronovolaan 5, 2597 AX, The Hague, The Netherlands; 3 Department of Medical Microbiology, Leiden University Medical Center, Albinusdreef 2, 2333 ZA, Leiden, The Netherlands; 4 Department of Internal Medicine, Medical Center Haaglanden, Lijnbaan 32, 2512 VA, The Hague, The Netherlands; 5 Department of Medical Microbiology, Bronovo Hospital, Bronovolaan 5, 2597 AX, The Hague, The Netherlands; 6 Department of Internal Medicine, Groene Hart Hospital, Bleulandweg 10, 2803 HH, Gouda, The Netherlands; 7 Department of Internal Medicine, Spaarne Hospital, Spaarnepoort 1, 2134 TM, Hoofddorp, The Netherlands; 8 Department of Internal Medicine, Rijnland Hospital, Simon Smitweg 1, 2353 GA, Leiderdorp, The Netherlands; 9 Department of Internal Medicine, Diaconessenhuis Leiden, Houtlaan 55, 2334 CK, Leiden, The Netherlands

## Abstract

**Introduction:**

Guidelines recommend that two blood cultures be performed in patients with febrile urinary tract infection (UTI), to detect bacteremia and help diagnose urosepsis. The usefulness and cost-effectiveness of this practice have been criticized. This study aimed to evaluate clinical characteristics and the biomarker procalcitonin (PCT) as an aid in predicting bacteremia.

**Methods:**

A prospective observational multicenter cohort study included consecutive adults with febrile UTI in 35 primary care units and 8 emergency departments of 7 regional hospitals. Clinical and microbiological data were collected and PCT and time to positivity (TTP) of blood culture were measured.

**Results:**

Of 581 evaluable patients, 136 (23%) had bacteremia. The median age was 66 years (interquartile range 46 to 78 years) and 219 (38%) were male. We evaluated three different models: a clinical model including seven bed-side characteristics, the clinical model plus PCT, and a PCT only model. The diagnostic abilities of these models as reflected by area under the curve of the receiver operating characteristic were 0.71 (95% confidence interval (CI): 0.66 to 0.76), 0.79 (95% CI: 0.75 to 0.83) and 0.73 (95% CI: 0.68 to 0.77) respectively. Calculating corresponding sensitivity and specificity for the presence of bacteremia after each step of adding a significant predictor in the model yielded that the PCT > 0.25 μg/l only model had the best diagnostic performance (sensitivity 0.95; 95% CI: 0.89 to 0.98, specificity 0.50; 95% CI: 0.46 to 0.55). Using PCT as a single decision tool, this would result in 40% fewer blood cultures being taken, while still identifying 94 to 99% of patients with bacteremia.

The TTP of *E. coli *positive blood cultures was linearly correlated with the PCT log value; the higher the PCT the shorter the TTP (R^2 ^= 0.278, *P *= 0.007).

**Conclusions:**

PCT accurately predicts the presence of bacteremia and bacterial load in patients with febrile UTI. This may be a helpful biomarker to limit use of blood culture resources.

## Introduction

Urinary tract infection (UTI) is one of the most common infectious diseases. Fever in UTI typically represents the presence of acute pyelonephritis but it may also reflect prostatitis and/or the urosepsis syndrome [1,2]. Patients with febrile UTI generally present with mild illness in primary care but may rapidly develop a life-threatening condition, progressing into septic shock and multiple organ failure. The overall mortality rate of pyelonephritis is approximately 0.3%, but in bacteremic patients it can be as high as 7.5% to 30% [3,4]. In addition, bacteremia in UTI is associated with prolonged hospitalization and higher complication rates [5-7]. Given this spectrum of disease, clinicians are vigilant to identify bacteremia at a patient's presentation.

The incidence of bacteremia in patients with acute pyelonephritis has been reported to be roughly 20% [8-10]. Several studies have been conducted to identify predictive characteristics of bacteremia in patients with UTI [6,7,11,12]. However, no single clinical model has been used in practice because of its poor value in predicting bacteremia. The gold standard for detection of bacteremia remains the performance of at least two blood cultures to achieve sufficient sensitivity [13]. There are, however, practical limitations. First of all, it takes at least 24 to 48 hours to attain the culture result. Secondly, there may be a false positive result as contamination rates of up to 7% have been reported [14]. Furthermore, the implementation of the surviving sepsis campaign, which recommends the immediate initiation of broad-spectrum antibiotic therapy once septicemia is suspected, leads to an increase in the performance of blood cultures with lower yield, likely reflecting the obtainment of additional cultures after initiation of antibiotics [15,16]. Therefore, there is a need for strategies that guide clinicians and help reduce avoidable blood cultures and, by consequence, medical costs.

The biomarker procalcitonin (PCT) is a marker of systemic inflammation and thus it may help to predict bacteremia [17,18]. The aim of this study was to assess clinical characteristics and the PCT value to predict bacteremia in patients with febrile UTI.

## Materials and methods

### Study design and setting

We conducted a prospective observational multicenter cohort study. Eight emergency departments (ED) of 7 hospitals and 35 affiliating primary health care centers, serving one single area of the Netherlands, participated. Consecutive patients who presented with a diagnosis of febrile UTI, were considered for enrollment in the study. Recruitment took place from January 2004 through November 2008 but each centre started at different time points. The study was approved by the local ethics committees and all included patients gave written informed consent.

### Inclusion and exclusion criteria

Inclusion criteria were: age of 18 years or above, fever (defined as an tympanic temperature ≥38.0°C or a history of fever and chills within 24 hours before presentation), at least one symptom of UTI (dysuria, frequency, urgency, perineal pain, flank pain or costovertebral tenderness) and a positive nitrite dipstick test or leukocyturia as defined by a positive leukocyte esterase dipstick test or the presence of more than five leukocytes per high-power field in a centrifuged sediment. Exclusion criteria were current treatment for urolithiasis or hydronephrosis, pregnancy, hemo- or peritoneal dialysis, a history of kidney transplantation or known presence of polycystic kidney disease.

### Procedures and definitions

Clinical data and laboratory values were collected by qualified research nurses or the clinical investigators (CvN, TNB). Baseline data were collected within 24 hours of enrolment by a standardized questionnaire of the patient and reviewing the medical record. All patients were empirically treated with antibiotics according to local policy (oral ciprofloxacin 500 mg twice daily for outpatients and cefuroxim ± gentamicin intravenously for inpatients). Based on the culture results, hospitalized patients were subsequently switched to oral antibiotic treatment (first choice ciprofloxacin).

Blood cultures were obtained before commencement of antimicrobial therapy and were analyzed using local standard microbiological methods. At least two sets of 10 mL blood samples were taken and inoculated into aerobic bottles, which were incubated into an automated continuous monitoring system. In the Leiden University Medical Center (LUMC), the BACTEC 9240 (Becton Dickinson Diagnostic Instrument Systems, Sparks, MD, USA) was used, which monitors CO_2 _production every 10 minutes by means of a fluorescent signal. The bottles were loaded in the automated system once received at the laboratory. The time to positivity (TTP), defined as the time from the start of incubation to the start of the alert signal (as documented by the monitoring system), was recorded for each bottle of positive blood cultures. When multiple cultures were positive, the shortest TTP was selected for analysis. TTP was analyzed for *E. coli *positive blood cultures and confined to results in one center, the LUMC, as the TTP depends on the microorganism and the logistics of blood culture performance (for example, transport time from blood culture obtainment to incubator) [19].

Clean midstream-catch urine cultures were obtained before starting antimicrobial therapy and were analyzed using local standard microbiological methods. In case of a urinary catheter, the urine sample was collected from the port of the catheter. A positive urine culture was defined as bacterial growth over 10^3 ^CFU/ml urine or a bacterial monoculture over 10^2 ^CFU/ml urine in the presence of pyuria [20]. Urine cultures revealing growth of two or more different bacterial species reflecting mixed skin or gut flora were considered to indicate contamination [20].

Plasma EDTA blood samples were collected, centrifuged and stored at -80°C within two hours of patient enrolment. PCT levels were measured after the completion of all study enrolments, using a Time Resolved Amplified Cryptate Emission technology assay (TRACE^®^, Kryptor compact, PCTsensitive; Brahms AG; Hennigsdorf, Germany).

Bacteremia was defined as growth of any pathogen in the blood culture. The isolation of coagulase-negative s*taphylococci *from the blood culture was considered to indicate contamination and thus absence of bacteremia.

### Statistical analysis

Descriptive analysis included means or percentages with 95% confidence intervals (CIs) or medians and ranges, as appropriate. Missing values of categorical variables were considered to indicate the absence of that characteristic. This was applied for shaking chills (*n *= 66) and costovertebral tenderness (*n *= 18). Univariate analysis was performed using the Student's *t*-test or Mann-Whitney *U *test for continuous variables and Chi-square tests for categorical variables. Covariates found to be associated with bacteremia on univariate analysis at a level of significance *P *< 0.2 were eligible for inclusion in a multivariate logistic regression model using a backward selection procedure [21]. Measures for association were expressed as odds ratios (ORs) for disease with their 95% CIs for categorical variables. We tested the following three models: 1). A clinical model including clinical variables only; 2). A clinical model added with the PCT value; 3). A model based on PCT only. The predicted probabilities of bacteremia (*P*_bac_) in any patient for the different models were calculated by using the following regression equation: ln (*P*_bac _/(1- *P*_bac_)) = intercept + β-coefficient * variable, where the intercept and β-coefficient are obtained from logistic regression analysis. We constructed receiver operating characteristic (ROC)-curves for the different models using *P*_bac _as the test variable and bacteremia (yes/no) as state variable. The discriminative power and the diagnostic performance of the prediction models were compared by calculating the area under the curve (AUC) of the ROC-curve and by Nagelkerke's R^2^. In addition, for the clinical models, based on the β-coefficient, points were assigned for each predictor and different cutoff values were used to calculate corresponding sensitivity, specificity, positive and negative predictive values (PPV, NPV) and likelihood ratios for predicting bacteremia were calculated. For PCT, different cutoff values were tested, according to the instructions by the manufacturer for diagnosis of bacterial sepsis or lower respiratory tract infection; the cutoff value corresponding with a sensitivity of 95% and highest specificity was chosen for further analysis. A *P*-value < 0.05 was considered indicative for statistical significance. SPSS software (SPSS Inc., Chicago, Ill, USA; version 17.0) was used for statistical analysis.

## Results

### Patient characteristics and microbiological results

Of 728 patients screened for eligibility, 642 met the inclusion criteria and were included in the study of which 581 were evaluable with concurrent blood cultures and PCT measurements at baseline. Patients excluded from analysis because of missing blood culture or PCT value, were similar with respect to demographics and clinical features. The majority (75%) presented at EDs. The median age was 66 years, 38% were men and 52% had co-existing illnesses. Details of the baseline characteristics are listed in Table 1.

**Table 1:** Baseline characteristics of 581 patients presenting with febrile UTI.

Characteristic at presentation	All patients *n *= 581	Non-bacteremic *n *= 450	Bacteremic *n *= 131	*P*
Demographics				
Age, years, median (IQR)	66 (46 to 78)	63 (42 to 77)	74 (60 to 84)	<0.001
Male sex	219 (38)	163 (36)	56 (43)	0.175
Nursing home residency	29 (5)	20 (4)	9 (7)	0.262
Co-morbidity				
Any	301 (52)	226 (50)	75 (57)	0.156
Diabetes mellitus	96 (17)	65 (14)	31 (24)	0.012
Malignancy^a^	63 (11)	44 (10)	19 (15)	0.126
Urinary catheter^b^	45 (8)	30 (7)	15 (12)	0.093
Urinary tract disorder^c^	144 (25)	110 (24)	34 (26)	0.725
Immunocompromised	87 (15)	68 (15)	19 (15)	0.864
History, Signs and Symptoms				
Antibiotic UTI treatment^d^	167 (29)	119 (26)	48 (37)	0.023
Fever duration, hours, median (IQR)^e^	24 (12 to 53)	24 (12 to 48)	24 (12 to 72)	0.791
Altered mental status	46 (8)	27 (6)	19 (15)	0.002
Shaking chills	320 (55)	236 (52)	84 (64)	0.018
Costovertebral tenderness	352 (61)	283 (63)	69 (53)	0.035
Temperature, °C, mean ± SD	38.6 ± 1.05	38.5 ± 1.06	38.8 ± 0.99	0.001
MAP, mmHg, mean ± SD,	101 ± 17	102 ± 17	100 ± 19	0.479
HR, beats/minute, mean ± SD	93 ± 18	91 ± 17	98 ± 20	<0.001
PCT, μg/L, median (IQR)	0.41 (0.13 to 1.68)	0.25 (0.10 to 0.90)	2.29 (0.72 to 9.07)	<0.001

Data are presented as n (%) unless otherwise stated. IQR, interquartile range; SD, standard deviation; MAP, mean arterial pressure; HR, heart rate; PCT, procalcitonin. ^a ^Defined as any cancer except basal- or squamous-cell cancer of the skin that was active within the previous year. ^b ^Indwelling urethral catheter (*n *= 36), supra-pubic catheter (*n *= 7), intermittent urethral self-catherization (*n *= 2). ^c ^Defined as the presence of any functional or anatomical abnormality of the urinary tract. ^d ^Present oral antibiotic treatment for non-febrile UTI (that is, fever developed during UTI treatment). ^e ^Missing value in 81 patients.

Bacteremia was present in 131 (23%) patients: *Escherichia coli*, *n *= 104, (79%); *Klebsiella spp.*, *n *= 6, (5%); *Proteus spp.*, *n *= 5 (4%), *Pseudomonas aeroginosa*, *n *= 3 (2%); *Staphylococcus aureus*, *n *= 2 (2%); *Enterococcus spp.*, *n *= 2 (2%); and other, *n *= 9 (7%). Only sixteen patients (3%) had coagulase-negative s*taphylococci *in their blood culture; this was considered contamination. Bacteremic patients were significantly older, they significantly had more diabetes mellitus, shaking chills, or were pretreated for UTI; costovertebral tenderness was significantly less frequently present. On physical examination bacteremic patients more frequently had altered mental status, and they significantly had higher temperature and heart rate (Table 1).

Urine cultures were done in 559 (96%) patients and revealed the following: *E. coli*, *n *= 319 (57%); *Klebsiella spp.*, *n *= 25 (4%); *Pseudomonas aeroginosa*, *n *= 14 (3%); *Proteus spp*., *n *= 12 (2%); *Enterococcus spp*., *n *= 10 (2%); *Staphylococcus spp*., *n *= 12 (2%); other uropathogen, *n *= 21 (3%); contaminated, *n *= 77 (14%) and negative urine culture, *n *= 71 (13%). In those patients of whom no definite uropathogen was isolated, 69% had antibiotic UTI treatment during obtainment of the urine culture sample.

Results of con- and discordant blood and urine cultures have been described previously [22].

### Procalcitonin and microbiological outcome

**Figure 1:**
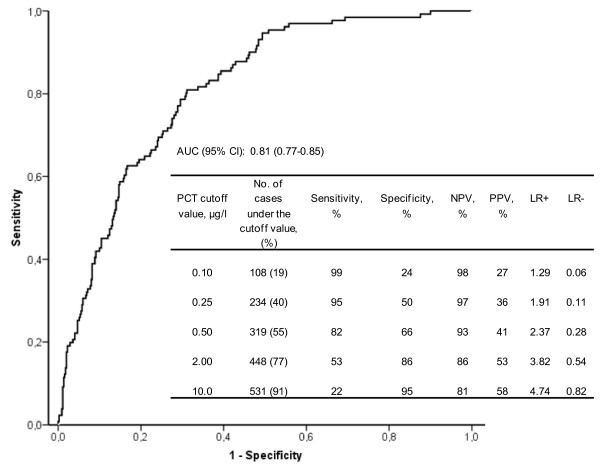
**Predictive value of procalcitonin (PCT) level for the diagnosis of bacteremia in 581 adults presenting with febrile urinary tract**. AUC, area under curve; ROC, receiver operating characteristic; NPV, negative predictive value; PPV, positive predictive value; LR+, positive likelihood ratio; LR-, negative likelihood ratio.

The AUC of the ROC-curve of PCT diagnosing bacteremia was 0.81 (95% CI: 0.77 to 0.85) indicating good discriminative power (Figure 1). The corresponding sensitivity, specificity, NPV, PPV and likelihood ratios of different PCT cutoff values are also outlined in Figure 1. A cutoff value >0.25 μg/l had a sensitivity of 95% and was chosen for further analysis in prediction modeling.

As the predictive value of PCT might have been influenced by the antibiotic treatment at the time of presentation in 29% of the patients, the analysis was also done separately. In patients on active antibiotic UTI treatment, bacteremia was present in 29% of the cases compared to 20% in those without antibiotic treatment. Corresponding AUC of the ROC-curve were 0.83 (95% CI: 0.76 to 0.89) and 0.80 (95% CI: 0.75 to 0.85), respectively, indicating that antibiotic treatment did not alter the predictive value of PCT with respect to bacteremia.

As undetectable PCT levels may be indicative of absence of bacterial infection we additionally tested whether a PCT value <0.06 μg/l was correlated with a negative urine culture. Indeed a PCT < 0.06 μg/l was associated with a lower rate of negative urine cultures, 11% versus 13% for PCT ≥ 0.06 μg/l, but this difference was not statistically significant (OR 0.8; 95% CI: 0.3 to 2.2, *P *= 0.821).

### Predictors of bacteremia

Clinical variables that were found to have an association with the presence of bacteremia with a *P*-value < 0.2 were entered as covariates into a multivariate logistic regression model. Then PCT > 0.25 μg/l was added as a variable in a second model and finally a univariate model of PCT > 0.25 μg/l was tested. This resulted in three different models (model 1, 2 and 3 respectively) as shown in Table 2. Older age, higher temperature and heart rate were significantly associated with bacteremia in the clinical model 1. When PCT was added to this clinical model (model 2), PCT appeared to be the strongest predictor (OR 14.7) for bacteremia, besides the significant clinical predictors temperature >38.6°C (OR 1.7) and diabetes mellitus (OR 1.8). The discriminative ability of model 2 with respect to Nagelkerke's R^2 ^was much better than the clinical model 1 (0.293 vs 0.145) but comparable with model 3 based on PCT only (0.252).

**Table 2:** Multivariate logistic regression models predicting bacteremia in 581 patients with febrile UTI.

	Multivariate OR (95% CI)	*P*-value	**R**^**2**^
**Model 1**			0.145
Age >65 years	2.4 (1.5 to 3.8)	<0.001	
Temperature >38.6°C	2.1 (1.3 to 3.3)	0.001	
Altered mental status	1.8 (0.9 to 3.5)	0.093	
Heart rate >100/minute	1.7 (1.1 to 2.7)	0.015	
Diabetes mellitus	1.6 (1.0 to 2.7)	0.063	
Shaking chills	1.5 (1.0 to 2.3)	0.052	
Antibiotic UTI treatment	1.5 (0.9 to 2.3)	0.085	
**Model 2**			0.293
Age >65 years	1.6 (1.0 to 2.5)	0.059	
Temperature >38.6°C	1.7 (1.1 to 2.7)	0.019	
Altered mental status	2.0 (1.0 to 4.2)	0.054	
Diabetes mellitus	1.8 (1.0 to 3.1)	0.035	
PCT > 0.25 μg/l	14.7 (6.6 to 32.6)	<0.001	
**Model 3 **(univariate)			0.252
PCT > 0.25 μg/l	18.0 (8.2 to 39.5)	<0.001	

UTI: urinary tract infection; OR: Odds Ratio; CI: confidence interval; PCT: procalcitonin; R^2^: Nagelkerke's R^2^.

Model 1 = Clinical model including all clinical variables of Table 1 with *P-*value < 0.2 in univariate analysis.

Model 2 = Model 1 + PCT > 0.25 μg/l. Model 3 = PCT > 0.25 μg/l only.

### Diagnostic value of prediction models

For each model we calculated the probability of bacteremia (*P*_bac_) for every individual patient with the equation as described above and compared the discriminative power of each model by constructing ROC-curves. Model 1, 2 and 3 had an AUC of ROC of 0.71 (95% CI: 0.66 to 0.76), 0.79 (95% CI: 0.75 to 0.83) and 0.73 (95% CI: 0.67 to 0.77), respectively.

In addition, we evaluated the diagnostic performance of each model in detecting bacteremia by measuring sensitivity, specificity, NPV, PPV and likelihood ratios. For model 1 and 2 we started with the most significant clinical predictor as indicated by the lowest *P*-value out of the multivariable analysis (Table 2) and then we stepwise added the next significant clinical predictor with increasing order of *P*-values. For each step, the corresponding sensitivity, specificity, NPV, PPV and likelihood ratios were calculated. In addition, the same was done in model 2 starting with PCT and then adding the clinical predictors. The results of this analysis are outlined in Table 3. Only model 2 and 3 including PCT as a predictor had a NPV >95% but model 3 (PCT > 0.25 μg/l only) had a better PPV. Thus the discriminative ability of PCT alone is better than PCT plus clinical predictors.

**Table 3:** Predictive value of different models predicting bacteremia in 581 adults with febrile UTI.

		No. patients without risk factor (%)	Sensitivity, % (95% CI)	Specificity, % (95% CI)	NPV, % (95% CI)	PPV, % (95% CI)	LR + (95% CI)	LR - (95% CI)
**Model 1**								
Risk factor	A	271 (47)	70 (62 to 78)	52 (46 to 56)	86 (81 to 89)	30 (25 to 35)	1.45 (1.25 to 1.68)	0.58 (0.44 to 0.75)
Risk factors	A, B	127 (22)	90 (83 to 94)	25 (21 to 30)	90 (83 to 94)	26 (22 to 30)	1.21 (1.12 to 1.30)	0.39 (0.23 to 0.66)
Risk factors	A, B, C	112 (19)	93 (87 to 97)	23 (19 to 27)	92 (85 to 96)	26 (22 to 30)	1.21 (1.13 to 1.29)	0.30 (0.16 to 0.57)
**Model 2**								
Risk factor	B	270 (46)	68 (60 to 76)	51 (46 to 56)	85 (80 to 89)	29 (24 to 34)	1.39 (1.21 to 1.62)	0.61 (0.48 to 0.80)
Risk factors	B, D	229 (39)	78 (70 to 85)	45 (40 to 49)	88 (83 to 92)	29 (25 to 34)	1.42 (1.26 to 1.61)	0.48 (0.34 to 0.67)
Risk factors	P, B	140 (24)	97 (92 to 99)	30 (26 to 34)	97 (92 to 99)	29 (25 to 33)	1.39 (1.30 to 1.49)	0.10 (0.04 to 0.27)
Risk factors	P, B, D	116 (20)	97 (92 to 99)	25 (21 to 29)	97 (91 to 99)	27 (23 to 32)	1.29 (1.21 to 1.37)	0.12 (0.05 to 0.33)
**Model 3**								
PCT > 0.25 µg/l		234 (40)	95 (89 to 98)	50 (46 to 55)	97 (94 to 99)	36 (31 to 41)	1.91 (1.73 to 2.11)	0.11 (0.05 to 0.22)

NPV, negative predictive value; PPV, positive predictive value; LR+, positive likelihood ration; LR-, negative likelihood ratio; A, Age >65 years; B, Temperature >38.6°C; C, heart rate >100/minute; D, diabetes mellitus; P, PCT > 0.25 μg/l. For Model 1 and Model 2 the corresponding sensitivity, specificity, NPV, PPV, LR+ and LR- are calculated using a cutoff value of ≥1 risk factor.

### Procalcitonin and time to positivity of blood culture

**Figure 2:**
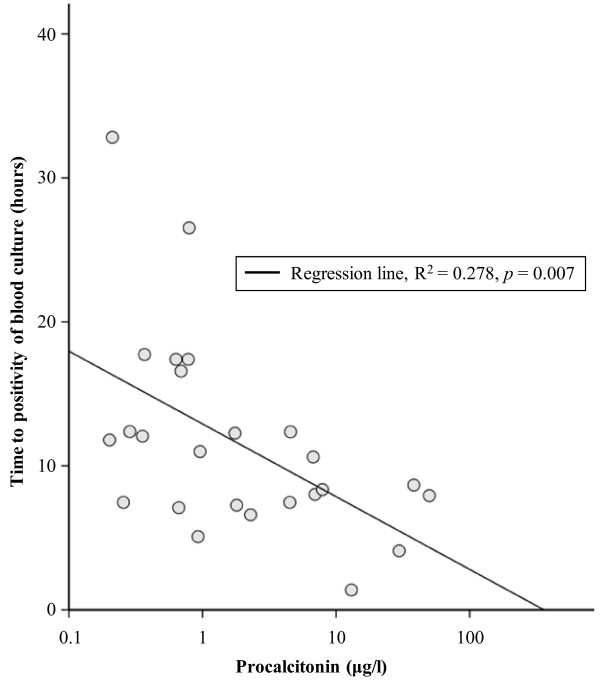
**Relation between procalcitonin level at presentation with *E. coli *urosepsis (*n *= 25) and time to positivity of blood culture**.

The TTP was available in 25 of 26 *E. coli *positive blood cultures. The mean TTP was 11.6 hours (range 1.3 to 31.4 hrs). Plotting TTP with the log value of PCT resulted in a significant linear correlation (R^2 ^= 0.278, *P *= 0.007), being the higher the PCT the shorter the TTP (Figure 2).

### Potential cost-savings of blood culture resources

We calculated potential cost-savings assuming two sets of blood cultures will cost $140 and the cost of PCT is $20 per measurement. In this cohort, using a preset PCT cutoff value of ≤0.25 μg/l would save 40% of blood cultures while still identifying 97% of bacteremias. Thus the potential saving in blood culture resources is ($140 times 0.40 minus $20) $36 per patient and $20.916 for the whole cohort of 581 patients.

## Discussion

In this study, we evaluated the ability of clinical and laboratory characteristics to predict bacteremia in adults presenting with febrile UTI. We found that PCT dichotomized around 0.25 μg/l, is a robust surrogate marker for bacteremia, whereas the actual PCT value reflects bacterial load in the blood stream. PCT might be applied to help guide and limit the use of blood culture resources.

We used a PCT cutoff value of ≤0.25 μg/l after having tested different standard cutoff values as has been advocated by the manufacturer's instructions to indicate absence or presence of sepsis or even absence or presence of bacterial infection as has previously been demonstrated in lower respiratory tract infections [23]. Compared to studies regarding PCT and bacteremia in infections other than febrile UTI, our diagnostic threshold was lower resulting in a higher sensitivity and lower specificity [17,18,24,25]. A recent study with similar design in patients presenting with community acquired pneumonia demonstrated highly similar findings [26]. In that study, a PCT value ≤0.25 μg/l would allow reducing blood cultures by 37% while still identifying 96% of bacteremias [26].

Using a PCT value ≤0.25 μg/l, we demonstrate a 40% reduction of blood cultures in our study population while still identifying 97% of bacteremias. Using PCT as a decision rule to guide taking blood cultures in febrile UTI would thus likely to be cost-effective. Moreover, it might prevent false-positive blood cultures and costs of associated medical consultations. However, other laboratory values that might routinely be measured in patients presenting with febrile UTI such as C-reactive protein (CRP) and the erythrocyte sedimentation rate (ESR) could also be indicative for the presence of bacteremia. In this study, CRP and ESR were measured in a subset of ED patients when indicated by the attending physician. Both were significantly associated with bacteremia but had very limited diagnostic ability compared to PCT (see Additional file 1). This is like other studies that did not recommend the use of CRP and ESR for diagnosing bacteremia [24,25].

The clinical characteristics associated with the presence of bacteremia comprise two categories. One comprises clinical signs which are a result of the host's response to bacterial components and cytokines elicited by the local infection and possible systemic expansion (that is, chills, confusion, temperature >38.6°C, heart rate >100/minute) and the other category includes host-related risk factors for a complicated clinical course of disease such as older age and diabetes. All these clinical factors were found to be associated with bacteremia in previous studies in patients with UTI [6,7,11,12]. Similar to previous reports on smaller cohorts, we were not able to accurately predict the presence of bacteremia based on clinical characteristics only. Likely, this can be explained in part by the relatively old study population (median age 66 years) as various related coexisting illnesses might result in heterogeneous symptoms of bacteremia [27].

A relationship between PCT and TTP of the blood cultures has indirectly been suggested in the setting of discriminating blood contamination from bloodstream infection due to coagulase-negative *staphylococci *[28]. However, to our knowledge a direct relationship between PCT and the TTP of the blood culture in gram negative bacteremia has not been addressed previously. As the majority of bacteremic UTI is caused by gram negative microorganisms, we hypothesized that the bacterial load likely reflects the level of free lipopolysaccharide and thus the level of endotoxemia, which is correlated with the PCT value [18]. The TTP of the blood culture that depends on the rate of carbon dioxide production by the microorganisms can be used as a surrogate for systemic bacterial load, and we, thus, analyzed its correlation with PCT [29]. Because the TTP depends on the microorganism and the logistics around blood culture obtainment, we decided to analyze this for *E. coli *bacteremias of one center only [19]. We found a significant loglinear relationship between PCT value and TTP that supports biological plausibility between PCT value and the bacterial load of infection. Probably a similar phenomenon is indirectly illustrated by studies in lower respiratory tract infection that demonstrated that a low PCT value reflects a self-limiting disease that does not require antibiotic treatment while higher PCT values are associated with complicated outcome [30,31]. However, it should be emphasized that in this study low PCT levels were not indicative of absence of urinary tract infection. Hence, all patients included in this study received antimicrobial treatment. Therefore, additional studies are needed as to whether PCT might be of value in guiding antibiotic treatment of UTI and decision upon hospitalization as non-bacteremic patients are likely to be good candidates for outpatient treatment. In this respect, the results of a recent study are not promising as they do not support the use of PCT in helping guide physicians in deciding about hospitalization in patients with acute pyelonephritis [32]. This is in accordance with a smaller study demonstrating that PCT was not correlated with adverse outcome of acute pyelonephritis [33]. Interestingly, this latter study also showed significant higher PCT levels in bacteremic patients compared to nonbacteremic patients.

Our study has several strengths. First of all, we prospectively included consecutive patients with febrile UTI at multiple sites at primary care and ED setting. Thus, our study population reflects the broad population of routine clinical practice. Secondly, we were able to achieve blood culture and PCT results in over 90% of the study population. Furthermore, the rate of bacteremia was 23% indicating that many patients suffered the urosepsis syndrome [2]. Recommended by sepsis guidelines, all such patients require blood cultures before the initiation of antibiotic treatment [16]. Yet, using PCT ≤ 0.25 μg/l as a decision rule would have resulted in a 40% reduction of blood culture utilization, with 3% loss of detection of bacteremia. The relation between PCT and TTP supports previous suggestions in other infections that PCT may serve as a predictive biomarker for degree and severity of bacterial invasion.

There may, however, also be some limitations. Almost 30% of the patients did use antibiotics at the time of presentation as fever apparently developed during treatment of a nonfebrile UTI, for example, cystitis. This may have led to false negative blood cultures and could contribute to a relative low specificity of PCT in diagnosing bacteremia. However, antibiotic pretreatment for cystitis in The Netherlands usually concerns nitrofurantoin, a drug that is unlikely to affect bacteremia in UTI. Consistent herewith, pretreatment was associated with a higher chance of bacteremia and this suggests that antibiotic pretreatment did not skew our results towards negative blood cultures. Nevertheless, this still does not exclude the possibility that the rate of bacteremia may reflect an underestimate. Another limitation might be the measurement of PCT values that was done afterwards. Though the frozen storage of blood sample does not influence its PCT value, the measurement of PCT in routine clinical practice might be different [34]. Furthermore, when used to limit the use of blood cultures, a quick result of PCT, preferably by a readily available point-of-care assay, is mandatory for practical reasons.

This study might have consequences for the current practices on EDs as implementation of a PCT strategy likely is a cost-effective way to avoid taking blood cultures with a very low chance of yielding a positive culture. Moreover, besides in febrile UTI, this also seems to hold for patients presenting with community acquired pneumonia [26]. Taken together, these studies suggest that in the majority of patients presenting with febrile illnesses at ED, being either respiratory or urinary tract infections, medical diagnostic costs can be reduced. However, it should be highlighted that additional validation studies are needed, as the in- and exclusion criteria applied in this study might limit its generalizability to other settings and special patients groups. Furthermore, implementation studies addressing its cost-effectiveness are needed before the widespread use of PCT guidance on doing blood cultures in routine clinical practice can be recommended.

## Conclusions

We conclude that PCT accurately predicts the presence of bacteremia and its bacterial load in adults with febrile UTI. A PCT value ≤0.25 μg/l sufficiently rules out bacteremia in febrile UTI and may be used to help guide efficient use of blood culture resources.

## Key messages

• According to sepsis guidelines, blood cultures should be drawn to help diagnose bacteremia in case of febrile UTI, but the usefulness and cost-effectiveness of this practice have been questioned.

• This study confirms that bacteremia in febrile UTI can neither be predicted nor ruled out by bedside available clinical parameters.

• A low value (≤0.25 μg/l) of the biomarker procalcitonin (PCT) sufficiently rules out bacteremia in febrile UTI.

• Implementation of PCT into clinical practice with the aim to limit avoidable blood cultures is likely to be cost effective.

• In case of bacteremia the level of PCT appeared to be a marker of the bacterial load. Whether this might have implications for the dosage and length of antibiotic treatment awaits further studies.

## Abbreviations

AUC: area under curve; CFU: colony forming unit; CI: confidence interval; CRP: C-reactive protein; ED: emergency department; ESR: erythrocyte sedimentation rate; LR: likelihood ratio; NPV: negative predictive value; OR: odds ratio; PCT: procalcitonin; PPV: positive predictive value; ROC: receiver operating characteristic; TTP: time to positivity; UTI: urinary tract infection.

## Competing interests

The authors declare that they have no competing interests.

## Authors' contributions

JWW, CN and JTD were responsible for the original design. CN, JWW and JTD were the guarantors. CN and TNB were responsible for data management, carried out the statistical analysis and wrote the initial draft supervised by JTD and JWW. CN, TNB, JWW, GHG, TK, GHWL, NMD, HCA and EMS were involved in patient recruitment and data collection. JWW, EJK, MJB, GHG, TK, GHWL, NMD, HCA and EMS critically revised the manuscript. All authors contributed to and approved the final version of the manuscript.

## Supplementary Material

Additional file 1 **Comparison of procalcitonin with C-reactive protein and erythrocyte sedimentation rate in predicting bacteremia in adults with febrile urinary tract infection**. Results of a subset of patients with febrile UTI with additional laboratory values available. Click here for file
