# Supplementary material for: Procalcitonin reflects bacteremia and bacterial load in urosepsis syndrome: a prospective observational study
Source: Crit Care. 2010 Nov 17;14(6):R206. doi: 10.1186/cc9328 (PMC3220019; doi:10.1186/cc9328)
Supplement: Additional file 1 — Comparison of procalcitonin with C-reactive protein and erythrocyte sedimentation rate in predicting bacteremia in adults with febrile urinary tract infection. Results of a subset of patients with febrile UTI with additional laboratory values available. [file cc9328-S1.DOCX]

**Additional file 1.** Comparison of procalcitonin with C-reactive protein and erythrocyte sedimentation rate in predicting bacteremia in adults with febrile urinary tract infection.

**Results**

In a subset of patients presenting with febrile UTI at emergency departments (ED), the C-reactive protein (CRP) and erythrocyte sedimentation rate (ESR) were measured as indicated by the attending physician using local standard procedures. This was done in 246 of 581 patients (42%). Of note, in the Netherlands over 90% of patients with febrile UTI presenting at the ED are referred by a primary care physician according to the guideline of the Dutch College of General Practitioners on the management of UTI that recommends referral to the hospital in case of pregnancy, failure of oral antibiotic treatment or suspected deterioration to severe sepsis.

Bacteremia was present in 72 (29%) patients. The median CRP in non-bacteremic patients was 106 mg/l [IQR: 55-195] compared to 166 mg/l [IQR: 60-276] in bacteremic patients (*p* = 0.006, Mann-Whitney *U* test). The median ESR in non-bacteremic patients was 39 mm/hr [IQR: 20-66] compared to 45 mm/hr [IQR: 29-73] in bacteremic patients (*p* = 0.047, Mann-Whitney *U* test).

The diagnostic performance of these laboratory values in predicting bacteremia compared to procalcitonin (PCT) is outlined in Figure S1. Though both CRP and ESR were significantly associated with the presence of bacteremia, the discriminatory power of PCT was significantly better.

As this is a subset of patients, the AUC of ROC of PCT for predicting bacteremia is slightly different from the AUC ROC calculated on the whole cohort (0.78 versus 0.81).


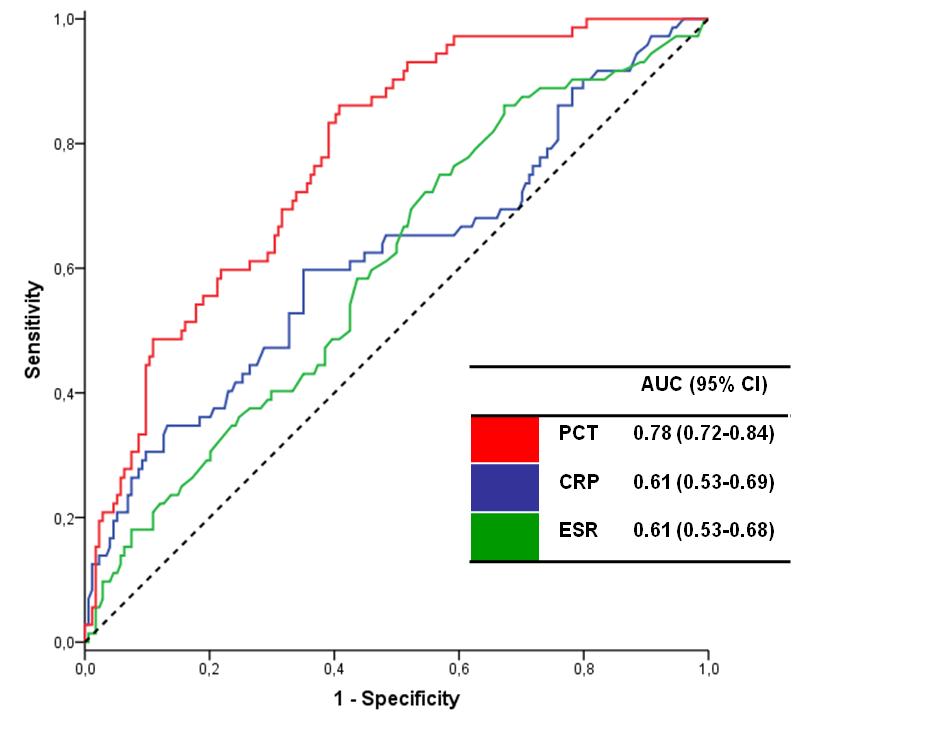


**Figure S1.** Receiver-operating characteristic curves of different laboratory values predicting bacteremia in 246 adults referred to emergency department because of febrile urinary tract infection (AUC: area under curve; PCT: procalcitonin; CRP: C-reactive protein; ESR: erythrocyte sedimentation rate).
